# Academic and healthcare efforts from Cessation to complete resumption of professional football tournaments during COVID-19 pandemic: A narrative review

**DOI:** 10.1016/j.heliyon.2023.e22519

**Published:** 2023-11-18

**Authors:** Naushad Ahmad Khan, Ayman El-Menyar, Mohammad Asim, Sameer Abdurahiman, AbdulWahab Abubaker Al Musleh, Hassan Al-Thani

**Affiliations:** a Department of Surgery, Trauma &Vascular Surgery, Clinical Research, Hamad General Hospital, Doha, Qatar; b Department of Clinical Medicine, Weill Cornell Medical College, Doha, Qatar; c Clinical Information Systems (CIS), Hamad Medical Corporation, Doha, Qatar; d Department of Surgery, Trauma and Vascular Surgery, Hamad General Hospital, Doha, Qatar

**Keywords:** Football, Coronavirus disease 2019, Pandemic, Risk-mitigation, FIFA world Cup 2022, Healthcare

## Abstract

The Coronavirus disease 2019 (COVID-19) caused by the SARS-CoV-2 virus led to over 626 million infections and 6.5 million deaths worldwide and forced to cancel or postpone several sporting events. Effective control techniques are therefore urgently required to avoid COVID-19 spread at these local and global events. This narrative review addressed the healthcare and research efforts on the intersections between COVID-19 and major professional sports leagues worldwide, with special reference to the FIFA World Cup football 2022. This explained how the broader transformation of COVID-19 from being a potential risk to an urgent pandemic public health emergency, caused the world of Football to halt between February and March 2020. This review could add to the growing literature on the importance of scientific research in understanding the relationship between mass sports events and COVID-19 trajectory, concerning studies conducted globally and particularly for the recommencement of major professional football competitions. The information outlined in the article may help sports organizations understand the risks associated with sports and their settings and improve their preparedness for future events under unprecedented circumstances. There were tremendous global healthcare and research efforts to deal with this unprecedented pandemic. The successful FIFA World Cup football tournament was an indicator of the success of these efforts**.**

## 1 Introduction

The COVID-19 pandemic has significantly affected all recreational, amateur, and major sports, leading to the cancellation and suspension of many professional sports competitions worldwide during the first half of 2020 [[1], [2], [3]]. With the imposition of risk mitigation measures such as social distancing, sporting activities were substantially hindered, even if played outdoors, and this changed the global sports landscape [4,5]. Because of the disease's novelty, lack of specialized mitigation strategies (like mass vaccination, which was rolled out widely from mid-January 2021) and effective treatment options, many preventive actions were undertaken around the world to minimize transmission of disease, such as stricter guidelines on social distancing; hand hygiene; the mandatory face masks [6], lockdown measures [7], travel restrictions [8], quarantine home isolation [9], and contact tracing [10].

During the COVID-19 epidemic era, there has been growing debate about whether professional Football should be resumed with spectators [11]. Football is known for its fervent supporters, crowded stadiums, and proximity between shouting and cheering fans, all of which might increase the transmission of SARS-CoV-2 infections via airborne particles and droplets [[12], [13], [14]]. These professional international football events with foreign competitors provide a public spectacle [12] while having significant implications for the host nation's social, political, economic, and public health domains, with an added risk of transmission of the infectious disease [1,15,16]. As a result, pandemics like COVID-19 have raised the stakes for determining the consequences of conducting major sporting events like Football. The football community throughout the world has worked diligently over the past years to discover answers for the safe recommencement of sporting events during the COVID-19 pandemic, and the great sports freeze finally appears to be thawing [17,18]. Sports leagues are marching ahead with plans to restart in a phased manner worldwide [11,[19], [20], [21], [22]]. In this regard, Qatar was the first country which resume professional sports events, including Football, successfully and safely during the initial stages of COVID-19, when the stakes were high for the possible spread of COVID-19 infections due to the non-introduction of vaccines [[22], [23], [24]].

Nevertheless, we now can evaluate the strategies and actions implemented, as well as the scientific study conducted by the state of Qatar, which has resulted in the successful restoration of professional football tournaments [11,[25], [26], [27]]. Moreover, the efficacy of these policies and details of actual data have been explored and published so far [11,22,26,28].

Notably, these data present the methodological underpinnings that describe the methods used to study COVID-19 infection and transmission among players and spectators at an event. It also reports infection rates during the pandemic, offering valuable insights for public policy to safeguard the health of players, spectators, and communities, highlighting effective safety measures. Opening up professional football games to spectators was one of the ticklish and challenging activities, that one would want to think about during the initial phase of the COVID-19 pandemic [29]. It has been noted that the host nation has had to reconcile concurrent expectations for in-person attendance and public safety concerns because of the consistent increase in COVID-19 community transmission throughout the second half of 2020 and the first part of 2021 [3,14,30,31]. However, all the major football leagues across the globe began their fall seasons without spectators [20,22,32]. The combination of political, economic, and sociological forces finally pushed several governments to ease attendance limits around major meetings and athletic events, allowing the people in stadiums (literally and figuratively).

However, rather than being dictated by league officials, the laws and guidelines controlling in-person attendance were largely set by state and local authorities, resulting in a patchwork of protocols entailing details such as physical distancing, use of face masks, spectator testing, and perhaps most importantly, maximum attendance [[3], [4], [5],^17^,^24^,^31^]. Several stadiums finally opened to a fraction of their original capacity, although the percentages fluctuated over time and across clubs. Nonetheless, because these gatherings had the potential to be super-spreader events, there was legitimate concern about exacerbating the already rising transmission of COVID-19 in the local population [1,14,33,34].

The current narrative review focuses primarily on major professional football leagues worldwide and in Qatar. It explores the emerging discourses on how and by what means sport's governing bodies responded and communicated to the COVID-19 crisis for the recommencement of professional football events between the early phase of the pandemic (March to May 2020). As it progresses, this review article highlights how global sports came to an abrupt and temporary standstill, and sport's governing organizations braced themselves to respond to the COVID-19 pandemic crisis within the sporting realm. Later, this article also emphasizes the appropriate responses in the form of strategic planning and the important role of sports research in emphasizing short-term adaption and long-term success during the COVID-19 crisis. We are responding to the recent calls for studies on the effect of the COVID-19 pandemic in the context of professional football involving mass gatherings from different perspectives [23,25,26]. This review aims to provide a summary of the existing academic literature to determine whether certain public health surveillance protocols provide insights into how the COVID-19 pandemic impacts large-scale professional football events, and potentially mitigate the associated risks.

### 1.1 Methodology

An extensive literature search was performed using PubMed, Embase, Web of Science, SCOPUS, and online databases. These databases were searched without restrictions to reclaim any publications related covid-19 and football between February 2020 and October 31, 2022. The search strategy included main strings viz “COVID-19”, “sport”, AND “Football” AND “spectators” as keywords for each string were used when building the search strategy. The combination of keywords used were (Covid-19 OR football OR sports) AND (infectious disease OR COVID-19 OR Coronavirus) AND (mass gathering events AND, spectators). To ensure that we didn't overlook any relevant publications/articles/commentary, we conducted an additional search on Google Scholar. Furthermore, we reviewed the reference lists of the chosen articles to identify any additional articles that might not have been included in the initial database search. Any literature/research article examining SARS-CoV-2 infections during COVID-19 from the above-mentioned search duration involving sports gatherings events i.e. football/soccer was considered eligible for inclusion. We excluded articles that were unrelated to viral infections or infectious diseases caused by non-viral pathogens, as well as, not related to football.

### 1.2 The global response during the early phase of COVID-19: “from Impending risk to immediate threat.”

This section details the time frame during which professional Football came to a halt (mainly in February and March of 2020) and gives a narrative of how the risk and threat of COVID-19 were addressed by the sport's governing organizations and other stakeholders. For instance, COVID-19 is a worldwide crisis that transcends national borders and is unselective and transnational [2,35]. Initially, responses to the initial COVID-19 epidemic were distinguished by "great uncertainty regarding the steps adopted by the various governments to prevent the pandemic spread" [30]. For perspective, COVID-19 was still regarded as an epidemic in January and February 2020, and it was uncertain precisely what consequence the crisis would bring on major sporting events and the rest of the world [36]. Still, different nations reacted to the epidemic quite differently despite international collaborative attempts.

As the virus spread, public health research started to comprehend the potential threat it posed to the global landscape of sports [36,37]. The Chinese Super League was halted on January 30, 2020, in China, where the virus was discovered for the first time [38]. It took three weeks for other nations to adopt the same extreme steps as China. However, in Europe, professional football leagues were not deferred until late February 2020 [30]. Italy, one of the hosts of Euro 2020, was hit by the virus before other nations [30]. Italian Serie A was therefore halted after 366 fatalities and 7375 confirmed cases were reported, and the Italian Serie A was played behind closed doors.

The state of Kuwait, a neighboring country of Qatar, canceled all soccer events on February 24, 2020, and Japan did the same the next day. The former had 191 cases at that time but no fatalities [39,40]. According to the statistics, there were 157 confirmed infections and one fatality. On February 28, 2020, Switzerland became the first country outside Asia to halt all soccer events [40]. As a result, just four nations worldwide have canceled or halted soccer as of the beginning of March 2020. Despite the transmission of the virus, travel restrictions, and other preventive measures put in place by different countries around the world during the first ten days of March, only 05 other football organizations suspended or canceled all events: Thailand suspended soccer events on March 3rd, Iran on March 4th, Italy on March 9th, Austria, and Portugal on March 10th, 2020 [40]. Some of these nations were already being severely affected by the epidemic.

However, the soccer matches were characterized by inconsistency because of the disparity in circumstances and responses across European nations. While RB Leipzig-Tottenham was played in Germany in front of a huge audience, Valencia-Atalanta was played in Spain behind closed doors [40]. The match between Paris Saint-Germain and Borussia Dortmund was played without spectators after the World Health Organization (WHO) declared COVID-19 to be a pandemic [30]. In contrast, Liverpool's match against Atletico Madrid was held in front of 52,000 spectators [41], illustrating the apparent nation-specific measures about sporting events and reports of infected athletes and managers [38].

The WHO labeled the fast-growing coronavirus outbreak a pandemic on March 11th, 2020, recognizing that the SARS-CoV-2 virus was likely to be transmitted worldwide [42]. In a way, WHO assessments marked the turning point when COVID-19 status transitioned from being a moderate risk to an absolute threat. Despite the centrality of this transition, the necessity to react to the danger and concrete threat of COVID-19 has not been limited to the health, scientific, political, economic, or educational spheres.

Subsequently, between the 12th and 13th of March, 41 nations postponed or halted all soccer-related activity, including leagues. Between the 14th and 18th of March, an additional 64 nations opted to stop professional soccer. In other words, soccer was suspended in 105 countries in only seven days [40]. On March 17th, UEFA, the European soccer governing body, announced the postponement European Championship. South America made a similar statement on the same day, shifting their Copa América to 2021 [37]. The UEFA Champions League began on March 13th, and the forthcoming matches were suspended. The 2020 African Nations Championship, which was slated to take place in Cameroon in April, was also rescheduled [37]. Almost the whole world's soccer leagues and other tournaments were suspended. A summary of some of the major global professional football events postponed and restarted or canceled during the initial phase (2020) of the COVID-19 pandemic is outlined in Table 1. These events demonstrated that soccer, like many other organizations worldwide, was hesitant to suspend operations until the continued infection spiraled out of control. The turning point occurred when the problem was classified as a pandemic. Other major professional soccer competitions were postponed in addition to the decision by the world's soccer organizations to shut down. Notably, these actions were taken to address perceived immediate threats as well as pandemic risk management [36,43].

**Table 1:** List of some major global professional football events postponed and restarted or canceled during the early phase (2020) of the COVID-19 pandemic.

Name of the Event	County	Initial Date (2020)	Due Date (2020)	Remarks
**Raiffeisen Super League**	Switzerland	Mar-02	Restarted, June 19	Matches are played behind closed doors without spectators
**Serie A**	Italy	Mar-09	Restarted, June 20	Matches are played behind closed doors without spectators
**La Liga**	Spain	Mar-12	Restarted June 11	Matches are played behind closed doors without spectators
**Premier League**	England	Mar-12	Restarted, June 17	Matches are played behind closed doors without spectators
**Liga NOS**	Portugal	Mar-12	Restarted, June 3	Matches are played behind closed doors without spectators
**Fortuna Liga**	Czechia	Mar-12	Restarted, May 23	Matches are played behind closed doors without spectators
**3 F Superliga**	Denmark	Mar-12	Restarted, May 23	Matches are played behind closed doors without spectators
**Scottish Premiership**	Scotland	March 12	Resumed August 1	Matches are played with a limited capacity of spectators
**Ligue 1**	France	Mar-13	Restarted on August 21	Matches are played without spectators
**Bundesliga**	Germany	Mar-13	Restarted, May 16	Matches are played behind closed doors without spectators
**PKO Ekstraklasa**	Poland	Mar-13	Restarted, May 29	Matches are played behind closed doors without spectators
**Premier Liga**	Russia	Mar-17	Restarted, June 19	Matches are played behind closed doors without spectators
**Tipico Bundesliga**	Austria	Mar-18	Restarted, June 2	Matches are played behind closed doors without spectators
**Eliteserien**	Norway	Season not started	Restarted, June 16	Matches played behind closed doors
**Allsvenskan**	Sweden	Season not started	Restarted, June 14	Spectators were allowed
**SPORTOTO SuperLig**	Turkey	Mar-19	Restarted, June 12	Matches are played behind closed doors without spectators
**2020 Copa America**	Brazil	June −12	Restarted, June 13	Matches are played behind closed doors without spectators 10 % Spectators allowed for final.
**2020 FIFA Club World Cup**	Qatar	December 2020	Restarted (1–February 11, 2021)	Matches are played with 50 % spectators
**2020 EFL Trophy Final**	UK	April-5	March 13, 2021	Spectators were allowed
**Euro Cup, 2020**	Different cities in Europe	12 June −12 July 2020	Restarted,11 June July 11, 2021	Spectators were allowed

### 1.3 Proactive scientific assessment and research for resumption of football

#### 1.3.1 - Global perspective

This section offers a scholarly reflection on how the major football leagues resumed the following suspension during the early part of the COVID-19 pandemic by adopting return to competition protocol, as well as extensive security measures worldwide and In Qatar. Several major football leagues opted to continue sporting activities in the latter stages of the COVID-19 epidemic in 2020.

A study by Meyer et al. detailed the effective resumption of the German professional football league (Bundesliga) without spectators, played during a time when the country had a low incidence of COVID-19 infections. They reported that twelve officials (0.7 %) out of 1702 routinely tested personnel tested positive during one of the initial rounds of PCR testing before the commencement of team training, whereas just two players returned positive at the end of the third round. The authors asserted that provided stringent hygiene precautions, including routine PCR testing, and professional football matches, could be undertaken safely throughout the COVID-19 pandemic [20].

Another study by the same group used commercial ELISA and a chemiluminescent immunoassay (CLIA) as screening assays to assess the seroprevalence in players and personnel from the German Bundesliga [44]. Participants underwent testing twice a week using PCR. In May and June 2020, the seropositivity among 1184 players and staff was 1.9 % and 2.1 %, respectively. Notably, all subjects tested negative for PCR during the research period. However, immunoglobulin G was discovered 8–10 times more often, showing a significant percentage of undiscovered infections among footballers [44].

Schreiber et al. evaluated the risk of COVID-19 transmission through interactions among on-field players in amateur, youth, and professional Football. Out of 1247 recognized matches, they detected 165 potentially affected players [45]. PCR testing did not find any evidence of transmission. The authors concluded that the likelihood of COVID-19 transmission on football fields is minimal and that football players' infections are probably not brought on by in-field-related activities.

A study published in Denmark evaluated the consequences of the controlled resumption of Football (soccer) strictly following a detailed protocol [21]. The study observed the number of players who tested positive for SARS-CoV-2 every week. In total, 6511 tests were done during the observation period, and the overall, COVID-19 infection rate was reported to be 0.06 % among the players.

Similarly, data from the US National Football League in 2020, Persian Gulf Pro leagues in Iran, Super League in Greece, SEC football games in the USA, and English Football League, all played without or limited presence of spectators, reported low incidence of COVID-19 infection among players and staffs [[46], [47], [48], [49], [50], [51], [52]]. These data suggested that the recommencement of football matches could be possible following strict compliance with the return to completion protocols.

However, some studies reported resumption of football matches could lead to an increase in COVID-19 cases in the community or the countries hosting the tournament [[53], [54], [55]]. This might be related to the fact that the competition took place at a time when the epidemiological trajectory of the COVID-19 pandemic was still evolving. With the COVID-19 immunization programs, governments were loosening their mitigating measures and permitting more cross-country migration. All these events also permitted people to return to the stadium. The mitigation of COVID-19 transmission strategies in stadiums is restricted by several parameters, such as the spectator's presence, with proximity, and the close contact among players on the field. Furthermore, the highly infectious and transmissible variant of concern (Omicron) was rapidly transmitted in Africa, Asia, and throughout Europe, with vaccinated people showing decreased protection against it [56,57]. A summary of published literature on football events initially postponed and resumed worldwide and their implications during the pandemic (2020-21) are outlined in Table 2.

**Table 2:** Detailed summary of global football events that were initially postponed and later resumed during the COVID-19 pandemic (2020-21).

Authors/Name of the Event Study period	Country	Study design	Positivity rate	SARS-CoV-2 strain in circulation	Precautionary measures	Conclusions
Meyer et al. [20] German Bundesliga (May–July 2020)	Germany	Prospective -Observational	0.7 % (12/1702) 0.74 % (8/1079) for Players 0.65 % (4/623) for officials	Alpha	Bundesliga Hygiene Protocol (BHP), PCR testing for SARS-CoV-2 RNA twice weekly, and antibody tests (on two occasions	Professional football training and matches can be carried out safely during the COVID-19 pandemic. This requires strict hygiene measures, including regular PCR testing.
Egger et al. [58] (August 2020–September 2020)	Germany	Retrospective- Observational	None	Alpha	Pre- and post-RT-PCR Testing	Very low risk of SARS-CoV-2 transmission during football matches.
Schreiber et al. [45] German first to third divisions Amateur and professional level (August 2020–March 2021)	Germany	Retrospective	13.2 % (165/1247)	Alpha and Delta	Routine and follow-up RT-PCR Testing	On-field transmission risk of SARS-CoV-2 in Football is very low. Sources of infections in football players are most likely not related to activities on the pitch.
Mack et al. [44] German Bundesliga (13 August 2020–22 May 2021)	Germany	Retrospective Observational	Not available	Delta	RT-PCR testing, Serological testing	Sero-prevalence with a high-quality diagnostic in Germany seemed to be around 2 %. The number of undetected infections was 8–10 times higher than in notification data. The quality of antibody assays is rather variable. Thus, results should ideally be confirmed at least by a second assay to prove IgG positivity.
Hassanmirzaei et al. [59]. (Persian Gulf Pro League & Iranian Hazfi Cup) 17 May-3 September 2020	Iran	Retrospective -Observational	17.8 % (144/805) 85 players (18.5 %) 59 staff/officials (17.05 %)	Alpha	Protocolized resumption of Football with RT-PCR testing	Repetitive PCR testing, symptom monitoring, case tracing, and strict hygiene protocols could aid the resumption of professional football competitions.
Hassanmirzaei et al. [49] (Persian Gulf League and Azadegan League) September–October 2020	Iran	Exploratory- observational	(2.3 %; 29/1243)	Alpha	Serological testing, RT-PCR testing	Inconsistency of results between the two tests; therefore, although the application of serological assays alone seems insufficient in diagnosing COVID-19, the findings are beneficial in the comprehension and management of the disease.
Pedersen et al. [21] Danish League May 19 -July 28, 2020	Denmark	Retrospective-observational	(0.53 %; 4/748) for players	Alpha	Testing protocol including Routine and follow-up RT-PCR Testing	The low incidence rate of SARS-CoV-2 no signs of a chain of infection. Controlled reopening of professional Football strictly adhering to a detailed protocol appears safe for the players.
Gualano et al. [54] 2020 football season, São Paulo July 4 December 21, 2020	Brazil	Retrospective cohort study	11.7 % (athletes) 7.2 % for staff	Alpha and Delta	Routine and follow-up RT-PCR Testing	High prevalence of SARS-CoV-2 infection despite weekly testing and other preventive measures after resuming Football, which coincided with the high prevalence of infection in the community during the same period
Cuschieri et al. [53] Euro Cup 11- June-11th of July 2021	11 countries across Europe	Retrospective-Observational	Not available	Delta	Protocolized resumption of Football with spectators, Vaccination, RT-PCR	A general increase in COVID-19 positivity trend in Europe was observed following a week of EURO2020 in cities hosting the matches.
Lopez et al. [47] National Football League (NFL) August 1, 2020–January 2, 2021	USA	Retrospective-Observational	55.5 % less among players compared to persons in the nearby counties	Alpha and Delta	NFL/NFLPA actively evolved protocols, Bubble concept, Routine RT-PCR testing weekly	55.7 % fewer observed COVID-19 infections among NFL players compared with simulated rates among persons of similar age in nearby counties. Implementation of NFL protocols was associated with lower infection rates among NFL players compared with the surrounding community. Robust testing and behavioral protocols support a safe return to sport and work.
Papagiannis D et al. [50] Super League May 2020 to May 2021	Greece	Prospective cohort study	0.57 % for players 0.27 % for staff	Alpha and Delta	weekly diagnostic testing (RT-PCR)	A low incidence of COVID-19 infection among professional footballers over a long follow-up period.
Basu et al. [52] English Football League 17June-26 July 2020	England	Retrospective-Observational	43 Participants (0.19 %) 18 players (0.08 %) 25 Staffs (0.11 %)	Alpha	Bi-weekly RT-PCR-based surveillance	With appropriate compliance, elite Football can continue safely during this pandemic. Protocols and compliance should be revised according to community prevalence and changes in viral transmission dynamics.
Dixon et al. [51] Southeastern Conference (SEC) football games September 26 -December 19, 2020	USA	Retrospective-cohort study	(11.6 %; 138/1190) players	Alpha	Surveillance by RT-PCR) 3 times per week, contact tracing	Active and vigilant surveillance can prevent the introduction of SARS-CoV-2 or similar threats into the gameplay, prevent game-specific exposures, transmission, and downstream infections, and reduce stress on public health systems.
Kurland et al. [55] The 2020/2021 National Football League (NFL) season March 11, 2020–1 March 2021	USA	Retrospective quasi-experimental study	COVID-19 cases/rates in the 14-day window in-county (rate ratio 1·36 [95 % CI 1·00–1·87], p < 0·01) for the 21-day window in-county (rate ratio 1·49 [95 % CI 1·21–1·83]	Alpha and Delta	Fans required compliance with basic safety and public health protocols, including face masks, mobile ticket entry , and a negative COVID-19 test.	Fan attendance led to episodic spikes in the incidence of COVID-19 infections in the 14-day window in-county, which had the venue and the surrounding counties in which fans traveled to attend. Games with less than 5000 fans did cause any spikes in the case rate. However, games played with over 20,000 fans generated significantly greater spikes in the case for the county in which games took place within the 21-day window, suggesting that return to sporting and other mass gathering events should be handled with extreme caution.
Taumi et al. [48] National Football League and National Collegiate Athletic Association Games August 29-December 28, 2020	USA	Cross-sectional observation study	Median (IQR) daily new COVID-19 cases in treatment group 26.14/100,000 residents on game day. For the control group, median daily new cases = 24.11 cases per 100,000 residents on game day.	Alpha and delta	General safety precautions (Hand Hygiene, face mask, and physical distancing), limited capacity of spectators, allowed	No significant increase in the daily COVID-19 cases/100,000 residents in counties where NFL and NCAA games were held with limited in-person attendance and not associated with substantial risk for increased local COVID-19 cases.

#### 1.3.2 Local perspective

Since being awarded the 2022 FIFA World Cup hosting rights, Qatar gained significant influence and has become a major force within global sports. During the previous decade, this Arabian Peninsula microstate has hosted many major sporting events and strengthened its worldwide footprint by investing in international sports through sponsorship deals, the acquisition of football teams, and the construction of cutting-edge athletic facilities. While much praise was given to the development as an alternative platform for Qatar (and the region in general) to operate on the global stage, the arrival of the COVID-19 pandemic raised concerns about this small country's operational preparedness in hosting this mega event in a safe environment.

More than two years of research and experience with COVID-19 increased our knowledge of the risks associated with sports settings involving large gatherings and have been proven to be a significant frontier for the resumption of professional football leagues in Qatar. Additionally, sports scientists have been brought in from all over the world to help the nation's top sports system flourish. The two foundations of this elite development plan are ASPITAR (Qatar's Orthopedic and Sports Medicine Hospital) and Aspire Sports Academy. Because of the rapid transmission of the COVID-19 pandemic, on March 14, 2020, the Qatar Olympic Committee (QOC) announced the suspension of all local sports activities, including professional football leagues, until March 29, 2020; later, it was extended until May 14, 2020, [[60], [61], [62]].

In May 2020, the government formed a task force consisting of public health scientists, sports physicians, and other stakeholders together, with the goal of resumption the football leagues in Qatar. Qatar became one of the first nations to establish an extended Bio-secure bubble system by adding several team participants for various sporting events with spectators in 2020 and continues to do so till now. It has successfully hosted Qatar Star League (QSL) 2020, West and East Zone tournaments of the Asian Football Confederation (AFC) Champions League, Amir Cup 2020, FIFA Club World Cup (FCWC) 2021, and FIFA Arab Cup 2021, with 30–50 to 100 % spectator's attendance (Table 3)

**Table 3:** Overview of football events hosted in Qatar during the pandemic (2020-21).

Tournaments	Number of participants	COVID-19 Variant	Spectators*	Sports resumption protocol	Precautionary measures
**Qatar Stars League (QSL)** [22] (June 8, 2020–September 2, 2020)	1337	Alpha	No	Return-to-competition	• Repeated PCR testing • Temperature check, Social distancing, Face mask (outside training and matches), Hand hygiene
**AFC Champions League (West region)** [63] (September 14 - October 3, 2020)	2184	Alpha	No	Bio-secure Bubble	• Protocolized PCR testing for all Bubble participants • Social distancing, Face mask (outside training and matches) • Bubble venue, transportation, and hotels • Protocolized results management
**AFC Champions League (East region) including the final** [11] (November 18, December 19, 2020)	3158	Alpha and Delta	Yes^†^	Bio-secure Bubble	• Same as above • Pre-testing all spectators • Spaced seating in the stadium • Social distancing, mask-wearing • Managed spectator entry process
**Amiri Cup** [25] (December 18, 2020)	2893	Alpha and Delta	Yes^††^	Bio-secure Bubble	• Same as AFC (East), including the final
**FIFA Club World Cup** [64] (February 1–11, 2021)	8192	Delta	Yes^†^	Bio-secure Bubble	• Same as AFC (East), including final
**FIFA Arab Cup** [26] (November 30- December 18, 2021)	6475	Delta and Omicron	Yes^†††^	General risk mitigation protocol, including mandatory vaccination with 100 % spectators.	Only vaccinated spectators are allowed. Social distancing, mask-wearing The managed spectator entry process Protocolized results management

*20%–30 % seating capacity; ^†^ spectators undergone rapid antigen testing; ^††^ spectators were not under bubble but had undergone antigen testing within 48 h of the event and also included individuals recovered from COVID-19 and underwent antibody testing as spectators; ^†††^ Vaccinated Spectators or Recovered spectators.

Qatar's resumption of football events was resumed with the Qatar Stars League (QSL), which was discontinued on March 16, 2020, due to the COVID outbreak. Later, it was resumed and completed successfully (June 8–September 2, 2020) by implementing a robust return-to-competition protocol. A prospective study by Schumacher et al. investigated the infectious and immunological status of 1377 players, club personnel, and match officials in QSL. This study was conducted in 2020, during the first wave of COVID-19 infection in Qatar, when the football season was curtailed due to the high incidence of infection [22]. A protocolized infection management approach comprising of preventative measures and routine PCR testing in conjunction with serology testing for immunity was implemented for this league. The study reported 85 subjects positive for COVID-19 (6.4 %), which was consistent with the infection rate of the general population over the same time. Furthermore, the majority of the infected players showed no symptoms, while the remaining had mild symptoms that did not warrant hospitalization. When preventive measures are in place, Football has a low risk of COVID-19 infection and severe illness even when players are in close contact.

Following that, another research conducted by the same group looked at whether SARS-CoV-2 contaminated random surfaces in football training facilities may be the source of COVID-19 transmission [28]. For this reason, random places such as training facilities, locker rooms, and medical and administrative areas were tested for SARS-CoV-2 for likely surface transmission (e.g., high-touch areas, cleaning equipment toilets, freezers, and pantries). According to the authors, none of the 103 swabs came out positive (ct value < 30). Sixteen swabs exhibited ct values between 35 and 40, whereas four samples had ct values between 30 and 35 (i.e., suggestive of low viral load). The other 83 samples were all negative. Even though some footballers were unwell, there was no indication of surface transmission in football club facilities when routine cleaning procedures and player monitoring were implemented.

Similarly, the Asian Football Confederation (AFC) Champions League was discontinued on March 4, 2020. Subsequently, with the control of SARS-CoV-2 transmission in August 2020, Qatar offered to host the AFC (West) championship league (a three-week event from September 14 to October 3, 2020) by implementing the Bio-secure Bubble protocol to get insight into the application of evidence-based strategies for the successful restart of the professional football league during the pandemic. Bio-secure bubble protocol entails adhering to strict protocols while in a hotel, traveling to venues, training sessions, sporting events, and visiting recreational areas. Isolation of players, staff, local organizing committee members, and other related personnel is required in a bio-secure bubble to ensure "no" or "limited" contact with people outside the bubble [[10], [11], [12]]. It also included protocolized screening and testing for COVID-19, secures transit, and regular disinfection of the tournament sites, (such as the media and training facilities) enabling Qatar to host West Zone matches for the 2020 AFC Champions League safely and successfully.

The tournament included 2184 participants, including 528 players, 388 team staff, and 1268 local staff. All participants undertook COVID-19 testing by reverse transcriptase polymerase chain reaction (RT-PCR) within 72 h of departure and upon entry in Qatar. Most participants tested negative (95.3 %), and (3.9 %) of the tests were inconclusive. During the entire tournament, the positivity rate was 2.7 % among all participants. Bio-secure bubble protocol operated in a structured and supervised setting presented a minimal risk of COVID-19 infection for hosting international football events [63].

The successful completion of the AFC West tournament under bio-secure protocol bolstered the organizers' confidence. It hosted several AFC Champions League (East) matches during November and December 2020. Al Musleh et al. [11] studied the impact of the recommencement of professional Football within a stringent Bio-secure bubble procedure and the impact of fans' participation during the tournament. Over 10,000 fans (30 % attendance) from the AFC Champions League (East) and the final game, as well as match officials, local organizing committee members, referees, hotel employees, and security personnel, participated in the study.

In the Bio-secure bubble, a total of 12,250 RT-PCR tests involving 3158 individuals were conducted. During the event, only five individuals (three local staff and two match officials) tested positive for COVID-19. No player was found to be positive for COVID-19. No one who tested positive needed hospitalization beyond symptomatic care; all were asymptomatic or had minor symptoms. The AFC (East) Champions League's total positive rate was 0.15%. A total of 10,320 fast Antigen tests for fans were conducted during the championship game; however, only one test resulted in COVID-19 positivity. The study concluded that, under the epidemiological conditions that existed between November and December 2020 in Qatar, the resumption of professional football with rigorous adherence to the Bio-secure bubble procedure, as well as the gradual return of a restricted number of fans, were not linked to an increase incidence of COVID-19 infections.

Following that, another retrospective-observational study from the same group [25] assessed the effects of resuming professional Football with fans and its effectiveness in preventing the transmission of COVID-19 infections in the local community. This event included rapid antigen and antibody testing as screening tools for spectators. A total of 16,171 fans underwent rapid antigen and serology testing and 15 were found to be positive (positivity rate = 0.12 %). Post-Amir Cup final, COVID-19-associated symptoms were observed in 1311 individuals (8.1 %). These spectators underwent RT-PCR testing, and a positivity rate of 0.42 % was reported. The authors concluded that the phased return of spectators to stadiums and the restarting of professional football games while strictly adhering to the bio-secure bubble procedure were both safe and did not contribute to the transmission of COVID-19.

The International Federation of International Football (FIFA), as the governing body of international Football, has the duty and mission to give relevant information and ideas to its member associations and other partners to minimize the impacts of the COVID-19 pandemic. The FIFA Club World Cup (FCWC, 2021) was the first competition held by FIFA since the start of the epidemic. This was originally scheduled for December 2020 in Qatar but was then pushed back to February 1–11, 2021, and then again to February 4–11, 2021. Massey et al. conducted prospective research to ascertain the transmission of COVID-19 among staff with the application of a bubble concept that included testing, hygiene, distance, and surveillance strategies such as risk-mitigation measures during the FIFA FCWC 2021 [64]. The study included all on-site staff. The competition included six teams and was conducted between 14 January and February 11, 2021. The tournament was conducted within a 'bio-secure bubble' protocol. All participants were required to have a negative RT-PCR test within 72 h of their arrival and were subjected to routine testing during the competition. The study included 70 participants and no person-to-staff transmission during the tournament was reported.

A cross-sectional study by Al-Thani et al. evaluated the incidence of COVID-19 and seasonal flu infections during the FIFA Arab Cup 2021, played with the full capacity of vaccinated or recovered spectators in Qatar [26]. The study also evaluated the attitude of spectators toward the recommencement of football events during the COVID-19 pandemic. The tournament included 16 teams and 32 matches over six different stadiums, all of which will be venues for the FIFA World Cup 2022.

Of the 10,000 spectators invited, 6475 participated in the study. For COVID-19, Respiratory Syncytial Virus (RSV), and influenza (A/B), there were 61 (0.9 %), 41 (0.6 %), and 11 (0.2 %) persons who tested positive, respectively. Overall, 33.9 % of individuals reported COVID-19-related symptoms of which 1.9 % were tested positive by PCR. The majority of spectators (94.3 %) were enthusiastic about restoring pre-pandemic status to sporting activities. This study found that the huge number of vaccinated spectators at the FIFA Arab Cup 2021 games did not result in a significant increase in the prevalence of local COVID-19 infections in Qatar. A summary of major professional football events hosted in Qatar during the COVID-19 pandemic is mentioned in Table 3.

The successful completion of the AFC championship league (West and East), the Amir Cup 2020, the FIFA FCWC World Cup 2021, and the FIFA Arab Cup 2021 marked the gradual return of football to be with fans during the COVID-19 pandemic in Qatar [11,25]. It was a watershed moment that highlighted Qatar's accomplishment, ability, and readiness to host a mass gathering-sporting event in the active phase of the COVID-19 pandemic.

Nevertheless, one overriding question, which also seems to be an important limitation of the prior studies, may contain bias that may have skewed their findings [11,19,20,22,26,48,53,[65], [66], [67]] because none of them addressed the different strains of SARS-Cov-2 when developing and implementing their various risk -mitigation protocols for resumption of professional Football. The different variants and subvariants of SARS-CoV-2 strains (Alpha, Delta, or Omicron) each have a distinct reproduction number (R0) value [68,69], with distinct transmissive and infective properties, which makes it difficult to compare the infection rates throughout a game or training session. The low risk of infection associated with the return of Football among players/spectators and the general population may be explained by the fact that most of the studies discussed in the current review have assessed infection rates during periods when Alpha and Beta strains were prevalent strains throughout the world (between early 2020 and the last quarter of 2021).

Therefore, one particular interest would be to do empirical studies evaluating the determinants of risks and infections and examine whether the emergence of more infectious new variants of concern (VOC) of SARS-CoV-2 has any impact on organizing mass gatherings events such as Football and on the health of players and spectators.

#### 1.3.3 Implications, challenges, and recommendations

More than 1.4 million fans and tourists attended the World Cup 2022 [70,71]. Few could have predicted that the COVID-19 epidemic, which initially appeared at the end of 2019 and the beginning of 2020, would last until 2021 and have a significant impact on professional sporting events throughout the world. The FIFA 2022 football World Cup tournament, comprising 32 foreign teams, was special considering the existing and evolving landscape of the pandemic caused by the introduction of VOCs [72]. To increase public vaccination trust, the football World Cup offered a unique opportunity for COVID-19 vaccine awareness and engagement with various sports organizations, health agencies, and governments globally. Despite the appearance of new COVID-19 variants, existing vaccines have been demonstrated to be effective in decreasing disease severity [73].

Nevertheless, hosting major international sports events has become increasingly difficult due to varying vaccination rates, dwindling immune status, and the advent of more contagious COVID-19 strains. As a result, hosting the FIFA Men's World Cup 2022 necessitated extensive preparation and interdisciplinary measures to avoid COVID-19 infections, including utilizing event publicity to increase vaccination use. Sports scientists and public health specialists adopted rigorous infection control strategies to reduce COVID-19 spread based on a series of studies done in Qatar from the early stages of the pandemic in 2020. Considering the pattern of the COVID-19 pandemic and the efficacy of vaccines, it was unlikely that the pandemic would pose a serious challenge to the FIFA World Cup 2022. While the state of Qatar might have proved through a series of pilot studies that the games could be held in a safe and infection-free environment during that phase of the pandemic, there were some major socioeconomic, scientific, and operational challenges.

Given the paucity of information about the long-standing durability of immunity in fully vaccinated individuals, the epidemiological landscape of COVID-19 for the FIFA World Cup football remained unknown. To some extent, immunization has stopped viral transmission; breakthrough infections and emerging novel strains have exacerbated the COVID-19 pandemic [[46], [74], [75], [76]]. The efficacy of booster doses and the rapid global spread of less severe variant (Omicron) offers promise for herd immunity [[73], [74]].

**Fig. 1:**
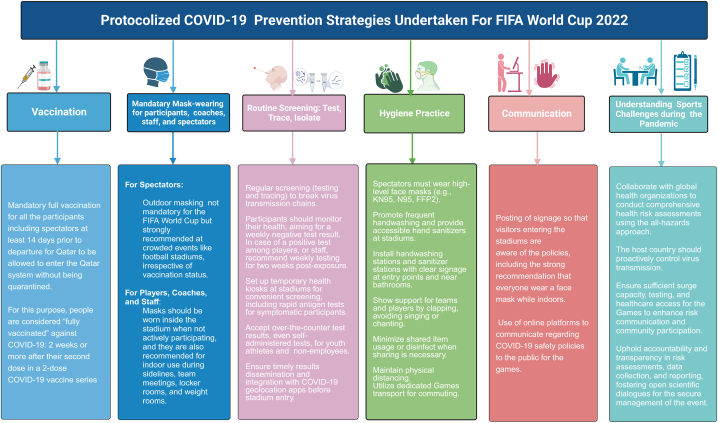
COVID-19 countermeasures for the FIFA Football World Cup 2022.

Notably, Qatar's previous experience with using an all-hazards approach that robustly recognizes various health hazards, conducting scientific risk assessment, risk mitigation, and ensuring greater transparency and effective communication was critical in managing the FIFA World Cup 2022 games in a safe environment. Moreover, Qatar has been actively involved in studying and implementing various infection control policies, including medical monitoring and risk mitigation protocols, over two and half years, which have underpinned the resumption of various Football events during the COVID-19 pandemic. Based on Qatar's past experiences hosting several football events during 2020–2021, the prevention and control strategies for mitigating the risk of COVID-19 transmission during the FIFA World Cup football 2022 are extrapolated as major recommendations in Fig. 1.

#### 1.3.4 The implication and relevance of artificial intelligence (AI) technology to other pandemics in global mass gathering events

The current global COVID-19 epidemic has presented an opportunity for the utilization of AI technology in the domains of public healthcare and other domains such as professional sports including football [58]. AI has made significant progress in its capacity to detect, screen, diagnose, and classify illnesses, assess biomarkers for predictive and prognostic applications, and develop strategies following diagnosis [59]. It may be employed to identify clusters of diseases, monitor cases, forecast future outbreaks, evaluate mortality risks, diagnose ailments, manage them, and reveal patterns that facilitate the examination of disease trends. Specifically, this technology may assume a crucial part in several facets of pandemic management in the realm of football events. The AI-driven algorithms may process timely identification and surveillance by analyzing comprehensive datasets that include health information, travel trends, and population dynamics to detect any indications of a disease epidemic. The scope of real-time monitoring including players, and spectators, may allow the timely detection of probable instances and the rapid deployment of containment protocols. AI-driven apps may play a crucial role in the domain of contact tracking, which had been the mainstay during the early outbreak of the COVID-19 pandemic to detect and isolate potential cases. These applications provide the rapid identification of persons who may have had close contact with an infected person, enabling their isolation and further testing.

Furthermore, it may facilitate the optimization of health screening procedures, enabling the automation of tasks like temperature monitoring and symptom evaluations inside stadium environments. Implementing automation guarantees that only those in a state of good health are granted access to matches, thereby effectively mitigating the potential risk of disease transmission [77].

The present review outlines valuable insights for making decisions based on data, which may include making schedule modifications, implementing restrictions on fan participation, or even deferring events if deemed essential. AI may further augment it effectively by handling various data sources such as player health records, crowd density, and infection rates, enhancing the efficiency of vaccine distribution strategies during a pandemic, particularly in the context of prioritizing those involved in football events, such as players, staff, and critical workers.

In situations when it is not possible to have in-person events, AI technology may be employed to facilitate virtual or augmented reality (VR/AR) experiences for fans. This would allow supporters to remotely participate in sporting events while following safety guidelines. The utilization of AI-powered communication technologies is expected to enhance communication and public awareness by delivering timely information to individuals, including fans and participants, about health recommendations, safety measures, and updates related to other future pandemics. Moreover, the huge amount of data generated so far from the COVID-19 pandemic can be integrated with an AI database which can assist in public health surveillance, real-time tracking of epidemic outbreaks, predicting current trends and future developments, routine updates and briefings from governmental agencies and organizations, as well as data on healthcare facility usage [78].

## 2 Conclusions

The COVID-19 pandemic had a remarkable influence on professional Football, highlighting the need to examine the sport-pandemic interface closely. This review makes a threefold academic contribution to the studies related to the resumption of Football during the COVID-19 pandemic by summarizing and extending pre-existing literature. First, it disaggregates the processes and sequentially breaks down the abrupt sporting freeze between February and May 2020. Second, it adds to the growing discourse on the significance of scientific research in understanding the relationship between sports during the COVID-19 era with special reference to studies conducted globally and in Qatar for the resumption of professional football events. Thirdly, It provides a summary of scientific knowledge of how sports institutional bodies representing the different nations and states have responded towards resolving the crisis, which might have also influenced public opinion on attending mass-gathering sporting events.

We are convinced that scientific researchers' contributions will be of immeasurable value in many ways that transition from publishing commentary or opinion pieces to the development of robust empirical and scientific protocols and publishing results that will pave ways and benchmarks of contextualizing how sport should be organized during pandemics.

## Ethics statement

No ethical approval is needed for this review article.

## Data availability statement

Data included in the text, tables, figures, and referenced in the article.

## Funding

This research received no external funding.

## CRediT authorship contribution statement

**Naushad Ahmad Khan:** Writing – review & editing, Writing – original draft, Visualization, Validation, Methodology, Conceptualization. **Ayman El-Menyar:** Writing – review & editing, Writing – original draft, Validation, Supervision, Resources, Data curation, Conceptualization. **Mohammad Asim:** Writing – review & editing, Writing – original draft, Visualization, Supervision, Resources, Methodology, Conceptualization. **Sameer Abdurahiman:** Writing – review & editing, Writing – original draft, Visualization, Resources, Methodology, Data curation, Conceptualization. **AbdulWahab Abubaker Al Musleh:** Writing – review & editing, Writing – original draft, Validation, Supervision, Data curation, Conceptualization. **Hassan Al-Thani:** Writing – review & editing, Conceptualization.

## Declaration of competing interest

The authors declare that they have no known competing financial interests or personal relationships that could have appeared to influence the work reported in this paper.
